# Estimated Daily Intake and Seasonal Food Sources of Quercetin in Japan

**DOI:** 10.3390/nu7042345

**Published:** 2015-04-02

**Authors:** Haruno Nishimuro, Hirofumi Ohnishi, Midori Sato, Mayumi Ohnishi-Kameyama, Izumi Matsunaga, Shigehiro Naito, Katsunari Ippoushi, Hideaki Oike, Tadahiro Nagata, Hiroshi Akasaka, Shigeyuki Saitoh, Kazuaki Shimamoto, Masuko Kobori

**Affiliations:** 1 National Food Research Institute, National Agriculture and Food Research Organization, Tsukuba, Ibaraki 305-8642, Japan; E-Mails: hn130901@as.seitoku.ac.jp (H.N.); kameyama@affrc.go.jp (M.O.-K.); izm@affrc.go.jp (I.M.); naito@affrc.go.jp (S.N.); Ippoushi@affrc.go.jp (K.I.); oike@affrc.go.jp (H.O.); 2 Department of Human Nutrition, Seitoku University, Matsudo, Chiba 271-8555, Japan; E-Mail: nagata@seitoku.ac.jp; 3 School of Medicine, Sapporo Medical University, Sapporo 060-8556, Japan; E-Mails: hohnishi@sapmed.ac.jp (H.O.); akasaka@sapmed.ac.jp (H.A.); 4 Sobetsu-cho, Usugun, Hokkaido 052-0101 Japan; E-Mail: sato.midori@town.sobetsu.lg.jp; 5 Sapporo Medical University School of Health Sciences, Sapporo, Hokkaido 060-8556, Japan; E-Mail: ssaitoh@sapmed.ac.jp; 6 Sapporo Medical University, Sapporo, Hokkaido 060-8556, Japan; E-Mail: simamoto@sapmed.ac.jp

**Keywords:** quercetin, onion, green tea, daily intake, Japanese, red leaf lettuce, asparagus

## Abstract

Quercetin is a promising food component, which can prevent lifestyle related diseases. To understand the dietary intake of quercetin in the subjects of a population-based cohort study and in the Japanese population, we first determined the quercetin content in foods available in the market during June and July in or near a town in Hokkaido, Japan. Red leaf lettuce, asparagus, and onions contained high amounts of quercetin derivatives. We then estimated the daily quercetin intake by 570 residents aged 20–92 years old in the town using a food frequency questionnaire (FFQ). The average and median quercetin intakes were 16.2 and 15.5 mg day^−1^, respectively. The quercetin intakes by men were lower than those by women; the quercetin intakes showed a low correlation with age in both men and women. The estimated quercetin intake was similar during summer and winter. Quercetin was mainly ingested from onions and green tea, both in summer and in winter. Vegetables, such as asparagus, green pepper, tomatoes, and red leaf lettuce, were good sources of quercetin in summer. Our results will help to elucidate the association between quercetin intake and risks of lifestyle-related diseases by further prospective cohort study and establish healthy dietary requirements with the consumption of more physiologically useful components from foods.

## 1. Introduction

Epidemiological studies have suggested that flavonoids, including quercetin, have a protective effect against cardiovascular diseases, cancers, and other chronic diseases [1,2,3,4,5,6,7]. Quercetin is a flavonol that is ubiquitously found in vegetables, fruits, and tea, as the glycosides [6,8]. The antioxidant activity [9,10,11], the anti-inflammatory effect [12], and/or other molecular mechanisms may prevent lifestyle-related diseases. Our previous study showed that a diet containing quercetin alleviated streptozotocin-induced diabetic symptoms in mice [13]. Quercetin was suggested to recover functions in both the liver and pancreas through oxidative stress reduction and the blockade of cyclin-dependent kinase inhibitor *p21*(*WAF1/Cip1*) (*Cdkn1a*) expression. Moreover, the consumption of a quercetin-rich diet alleviated obesity, hyperglycemia, hyperinsulinemia, and dyslipidemia in C57BL/6J mice that were fed a Western diet that was rich in fat, cholesterol, and sucrose [14]. Quercetin decreased oxidative stress and reducing peroxisome proliferator-activated receptor α expression that subsequently reduced the expression of genes related to steatosis in the liver. Recently, Dong *et al.* reported that quercetin suggested to suppress obesity-associated macrophage infiltration and inflammation through the adenosine monophosphate-activated protein kinase α1/silent information regulator 1 pathway in mice fed a high-fat diet [15].

Quercetin glycosides are mostly hydrolyzed and absorbed from the small or large intestines [6,8]. The physiological functions utilize the conjugated metabolites of quercetin in the plasma or other tissues, or the deconjugated aglycone in the specific tissues [8,16,17]. The intake of quercetin from dietary sources or supplements increases the plasma quercetin concentration [6]. Cao *et al.* reported that the mean intake over seven days of five flavonoids, including quercetin, was positively correlated to their corresponding plasma concentrations [18]. Ioku *et al.* showed that the quercetin glucoside content in onions did not decrease after frying or microwave heating [19]. Quercetin-4’-glucoside in onions was shown to transfer to the water during boiling without decomposition [19]. Thus, it is likely that the daily intake of food rich in quercetin increases the bioavailability of quercetin and contributes to the prevention of lifestyle-related diseases.

Sapporo Medical University has conducted a longitudinal population-based cohort study in the Tanno and Sobetsu area in Hokkaido. In the Tanno and Sobetsu study, central obesity assessed by waist circumference is shown to be useful for assessing the risk of type 2 diabetes [20]. Moreover, parental hypertension is shown to have an age-independent impact on the elevation of blood pressure, plasma glucose, and triglyceride levels, which may underlie the increase in cardiovascular events due to family history of hypertension [21]. Precise estimation of quercetin intake by subjects in the cohort study will help elucidate the relationship between quercetin intake and health indexes or risks of lifestyle related diseases. Sobetsu town is in an agricultural region and the major industry is fruit growing such as apples and cherries. The quercetin content of plant foods differs depending on the cultivars or cultivation conditions [22,23]. Therefore, in this study, to make a precise estimate of quercetin intake in the local residents, we first determined the quercetin content of the foods available in the markets in the Sobetsu area; we then estimated the daily quercetin intake by the residents in the Sobetsu town using a food frequency questionnaire (FFQ).

## 2. Experimental Section

### 2.1. Materials

One to three bags or bunches (approximately 0.2–1 kg bag^−1^ or bunch) of commonly eaten vegetables and fruits were obtained from three major farmer’s markets and the representative supermarket in the Sobetsu area (Sobetsu town, Toya town and Date city) during June, July, and December in 2013. Different bags or bunches of the same food were produced by the different farms. Autumn-planted onions grown on the main island and spring-planted onions grown in Hokkaido were available during summer and winter, respectively. Therefore, we determined the quercetin contents of onions and other vegetables and fruits commonly eaten in winter. The fruits and vegetables, except onions obtained in July, red leaf lettuce obtained in July, asupara-ra, and Chinese cabbage, were grown in the Sobetsu area. The edible parts, which were defined in the “Standard Tables of Food Composition in Japan (Fifth revised and enlarged editions),” in a bag or bunch were combined and reduced by sample division. Approximately 100–200 g of each food sample was frozen with liquid nitrogen, lyophilized, powdered in a grinder, and stored at −30 °C until the analysis. Approximately 150 g and 250 g of asparagus were cooked by boiling before the lyophilization respectively. Onions (200 g × 2) and green peppers (100 g × 2) were cooked by stirring before the lyophilization respectively. Green tea (200 g) and dried buckwheat noodles (400 g) were obtained from the representative supermarket in the Sobetsu area. Four grams of green tea leaves were infused twice with 100 mL of 80 °C water for 1 min. The green tea infusion was lyophilized, powdered, and then stored at −30 °C until the analysis. The dried buckwheat noodles were cooked by boiling before the lyophilization.

### 2.2. Determination of Quercetin Content

The quercetin content of each food sample was quantified by a high-performance liquid chromatography (HPLC) using the validated method of Watanabe *et al.* [24]. Briefly, quercetin aglycone was extracted from 200 mg of a freeze-dried food sample by hydrolysis with 12 mL of HCl solution (ethanol/water/HCl, 50:20:8, v/v/v) at 90 °C for 60 min, while shaking the sample solutions every 15 min. Five mL of green tea infusion was hydrolyzed with 12.5 mL of ethanol and 2 mL of HCl at 90 °C for 60 min. Each extract of vegetables, fruits, and tea was increased to 25 mL with methanol. Fifteen mL of each sample was filtered through a 0.45 μm polyvinylidene fluoride (PVDF) membrane filter prior to the HPLC analysis.

A Shimadzu HPLC Prominence that contained a degasser (DGU-20A3), binary pump (LC-20AB), auto-sampler (SIL-20AC), column oven (CTO-20A), and photodiode array detector (SPD-M20A) was used as the HPLC system. Ten microliter of each hydrolyzed food sample was applied to the HPLC column (Prodigy ODS (3), 5 µm, 100 A, 4.6 × 250 mm (Phenomenex, Torrance, CA, USA; Part No.00G-4097-E0)) and eluted with methanol/0.85% phosphoric acid (1:1, v/v) at a flow rate of 1.0 mL min^−1^ at 35 °C. The spectra were recorded from 200 nm to 500 nm, and quercetin was measured at 370 nm.

All food samples were determined in triplicate.

Quercetin content of foods are shown in Table 1. The quercetin content of cooked asparagus by boiling was 23.1 mg (100 g)^−1^ fresh weight. The quercetin contents of sautéed onions and green peppers were 7.7 and 7.5 mg per 100g fresh weight, respectively. The quercetin contents were slightly decreased by stir-frying. Among the foods we evaluated, spring-planted onions obtained in winter showed the highest quercetin content. The quercetin contents were less than the detection limit (0.07 mg g^−1^ of dry weight) in green and red shiso (*Perilla frutescens*), eggplants, welsh onions, cabbages, spinach, potatoes, buckwheat noodles, garland chrysanthemum, and Chinese cabbages.

### 2.3. Diet Survey

Sapporo Medical University has been conducting a cohort study called “The Tanno-Sobetsu study” since 1977. In the Tanno-Sobetsu Study, residents of two towns, Tanno and Sobetsu, in Hokkaido, Japan were recruited for annual medical examinations, including standard blood and urine tests. We recruited the study participants in the cohort of Sobetsu town. Sobetsu town is a rural area located in the island of “Hokkaido” in the north of Japan. Most participants in this cohort were middle-aged and elderly people and their life-style, obesity prevalence, blood pressure, blood glucose and lipid levels were similar to those in the results of national survey in Japan. Therefore, this cohort is considered to represent general Japanese population.

Two-day weighed food records (weight, servings, and portion size of food intake) were completed by eight volunteer housewives in July 2013. The FFQ, which asked about the frequency and portion size for 15 food items (onion, spinach, broccoli, potato, green pepper, asparagus, tomato, cherry tomato, cabbage, eggplant, red leaf lettuce, shiso (*Perilla frutescens*), cherry, buckwheat noodles, and green tea) was recorded by 570 residents aged 20–93 years; trained dieticians checked the information through interviews during July–August 2013. A FFQ which asked about the frequency and portion size for 14 food items (onion, spinach, broccoli, potato, green pepper, asparagus, tomato, cherry tomato, Chinese cabbage, garland chrysanthemum, red leaf lettuce, apples, buckwheat noodles, and green tea) was recorded by 60 residents aged 41–91 years; trained dieticians checked the responses through interviews in December 2013 in the same manner. The subjects were informed of the objective of the study and agreed to participate. The study design was approved by the Ethical Committee in Sapporo Medical University.

The intake of quercetin was estimated by the calculations of the food intakes and the quercetin content.

**Table 1:** Quercetin content in commonly-eaten foods in Japan.

Acquisition period	Food	Quercetin content
		(mg 100 g^−1^ FW or mg (100 mL)^−1^ *)
June–July 2013	Red leaf lettuce (*Lactuca sativa* L. var. *crispa*)	30.6
	Asparagus (*Asparagus officinalis* L.)	23.6
	Romaine lettuce (*Lactuca sativa* L. var. *longifolia*)	12.0
	Onion (*Allium cepa* L.)	11.0
	Green pepper (*Capscicum annuum* L.)	9.9
	Asupara-na (Brassica rapa)	4.3
	Cherry tomato (*Solanum lycopersicum*)	3.3
	Podded pea (*Pisum sativu* L.)	1.7
	Tomato (*Solanum lycopersicum*)	1.6
	Broccoli (*Brassica oleracea* var. *italica*)	1.6
	Cherry (*Prunus avium* L.)	1.2
	Green tea infusion	2.1*
	Welsh onion (*Allium fistulosum* L.)	N.D.
	Spinach (*Spinacia oleracea* L.)	N.D.
	Potato (*Solanum tuberosum* L.)	N.D.
	Red shiso^1^ (*Perilla frutescent* var. *crispa*)	N.D.
	Green shiso^2^ (*Perilla frutescent* var. *crispa*)	N.D.
	Eggplant (*Solanum melongena* L.)	N.D.
	Cabbage (*Brassica oleracea* L. var. *capitata*)	N.D.
	Dried buckwheat nudles (boiled)	N.D.
December 2013	Onion (*Allium cepa* L.)	41.9
	Red leaf lettuce (*Lactuca sativa* L. var. *crispa*)	10.3
	Apple (Fuji) (*Malus domestica* Borkh.)	2.3
	Broccoli (*Brassica oleracea* var. *italica*)	0.5
	Spinach (*Spinacia oleracea* L.)	N.D.
	Garland chrysanthemum (*Glebionis coronaria*)	N.D.
	Chinese cabbage (*Brassica rapa* var. *pekinensis*)	N.D.

N.D., not detected; FW, fresh weight. ^1^ *Perilla frutescens* var. *crispa* f. *crispa.* ^2^ *Perilla frutescens* var. *crispa* f. *purpurea*. Each food item was purchased 1–3 times and each sample was determined in triplicate. Values are expressed as mean of 1–3 samples.

### 2.4. Statistical Analysis

The statistical analyses were performed using GraphPad Prism 5 for Windows Ver. 5.04 (GraphPad Software, San Diego, CA, USA). The significance of the differences between groups was determined by the Mann–Whitney U test. We applied square root transformation to the daily quercetin intakes, which did not have a normal distribution, before assessing the correlation between quercetin intake and age. The association was established by the Pearson rank correlation test. A *p* value of <0.05 was considered statistically significant.

## 3. Results

### 3.1. Estimated Dietary Quercetin Intakes by Female Volunteers Using Two-Day Weighted Food Record

**Figure 1:**
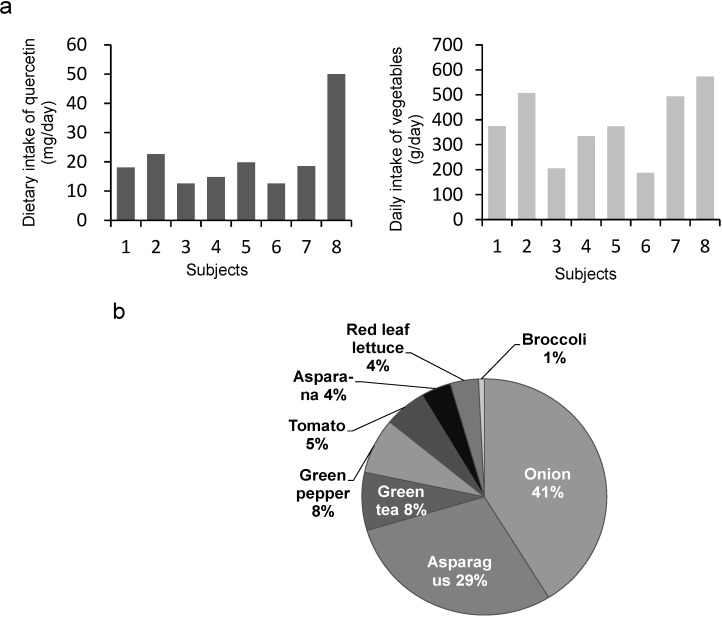
(**a**) Estimated intakes of quercetin and vegetables using two-day weighed food records of female volunteers. (**b**) Percentage contribution of foods to daily quercetin intake by the volunteers.

Two-day weighed food records were completed by eight volunteer housewives in Sobetsu town in Hokkaido in July 2013. Quercetin intake was estimated using the quercetin contents of foods obtained during June and July 2013. The estimated quercetin and vegetable intakes of each subject are shown in Figure 1a. The estimated daily vegetable intakes and quercetin intakes were 187–573 g day^−1^ and 12.6–49.9 mg day^−1^, respectively. The daily intake of quercetin was higher in subjects who consumed a large amount of vegetables. The average intake of vegetables and quercetin was 381 g day^−1^ and 21.5 mg day^−1^, respectively. The major sources of quercetin were onions, asparagus, green peppers, green tea, and tomatoes (Figure 1b).

### 3.2. Estimated Daily Quercetin Intakes of Residents by FFQ

The FFQ, which asked about the frequency and portion size of 15 commonly-eaten and quercetin-rich foods, was then completed by 570 residents of Sobetsu during July and August 2013. The subjects were 210 men and 360 women, aged 20–93 years. The average age was 65 years old. Quercetin intake was then estimated using the quercetin content of food obtained in June and July 2013.

**Figure 2:**
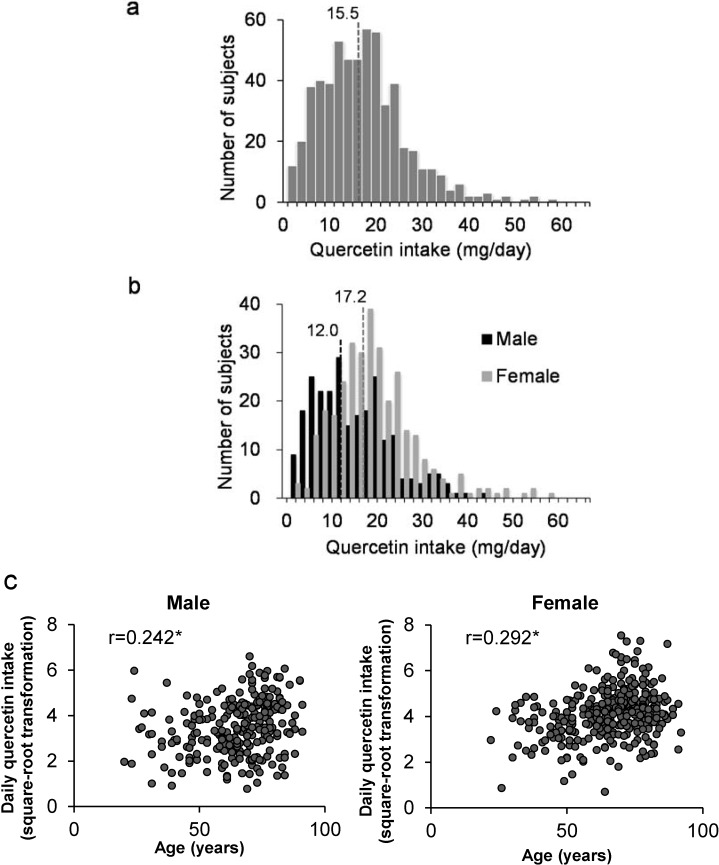
(**a**) Estimated daily quercetin intake by 570 residents of Sobetsu in Hokkaido using the FFQ during summer. (**b**) Estimated daily quercetin intakes by men and women using the FFQ during summer. Numbers in the figure show the median quercetin intakes by men and women. (**c**) Correlation between the daily quercetin intakes by men or women and their age. r, correlation coefficient; * *p* < 0.0001; Pearson correlation test.

**Figure 3:**
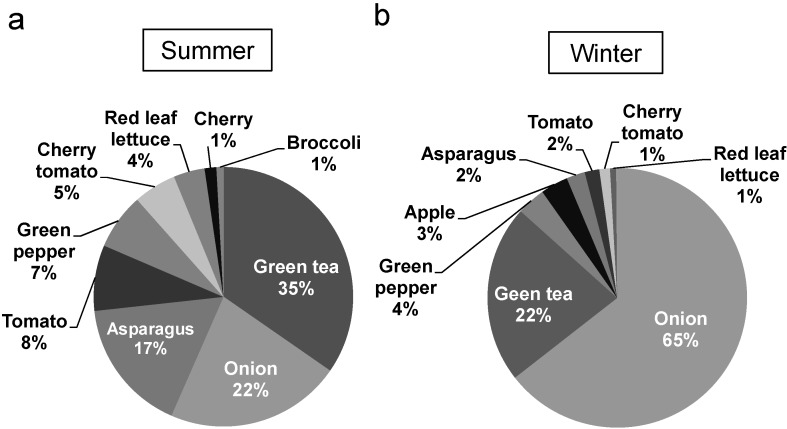
Percentage contribution of foods to the daily quercetin intake by residents of Sobetsu in Hokkaido during summer (**a**) and winter (**b**).

Figure 2a shows a frequency distribution chart of quercetin intake by the 570 residents. The estimated quercetin intake ranged from 0.5 to 56.8 mg day^−1^. The average and the median quercetin intakes were 16.2 mg day^−1^ and 15.5 mg day^−1^, respectively. Green tea was the main dietary source of quercetin, followed by onions, asparagus, tomatoes, and green peppers (Figure 3a). The average and median quercetin intakes by men and women were 13.8 and 12.0 mg day^−1^, and 18.3 and 17.2 mg day^−1^, respectively. The daily quercetin intake by women was significantly higher than the intake by men (*p* < 0.001) (Figure 2b).

The coefficients of correlation between quercetin intake and age were 0.242 and 0.292 for men and women, respectively (Figure 2b). Quercetin intake showed low correlations (*p* < 0.0001) with age for both men and women (Figure 2c).

**Figure 4:**
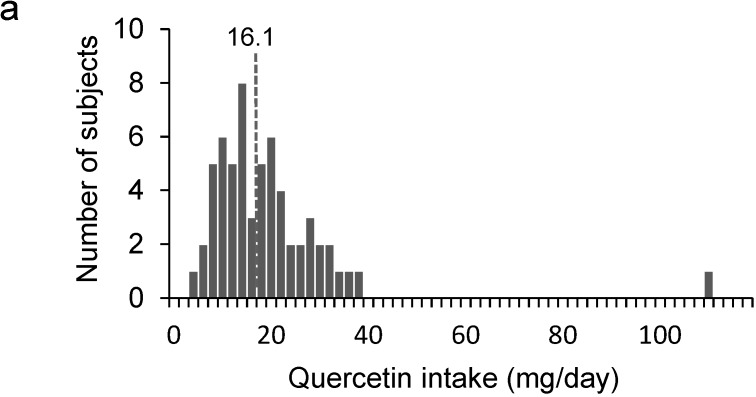

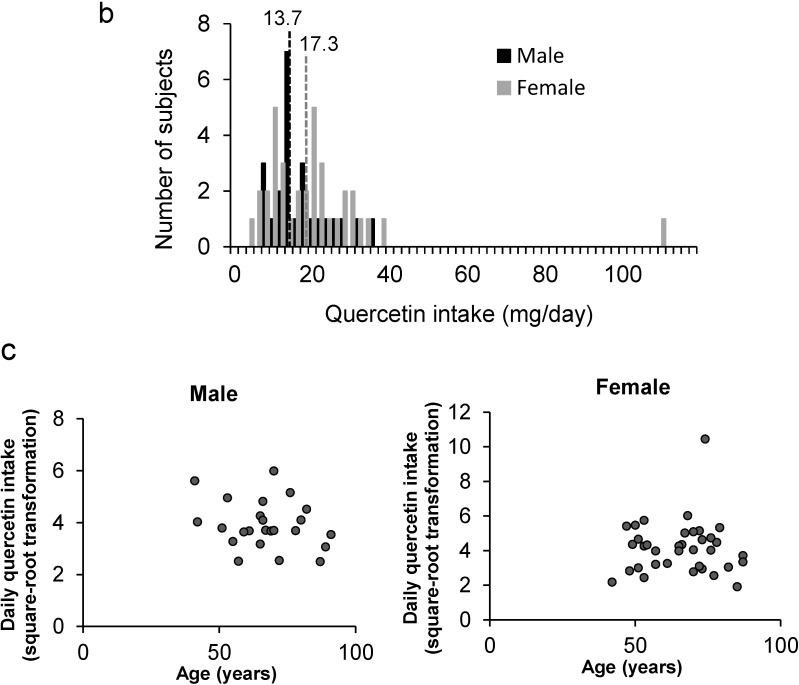
(**a**) Estimated daily quercetin intake by 60 residents of Sobetsu in Hokkaido using the FFQ during winter. (**b**) Estimated daily quercetin intakes by men and women using the FFQ during winter. Numbers in the figure show the median quercetin intakes by men and women. (**c**) Relationship between quercetin intakes by men or women and their age.

Another FFQ, which asked about the frequency and portion size of 14 commonly-eaten and quercetin-rich foods in winter, was completed by 60 residents of Sobetsu in December 2013. The subjects included 24 men and 36 women, aged 41–91 years. The average age was 66 years old. The quercetin contents of onions, red leaf lettuce, apples, and broccoli in December 2013 were used for estimating the quercetin intake. The estimated quercetin intake ranged from 3.7 to 109.1 mg day^−1^ (Figure 4a). The average and the median quercetin intakes were 18.3 mg day^−1^ and 16.1 mg day^−1^, respectively (Figure 4a). The estimated quercetin intakes in winter were not significantly different from those in summer. The average and median quercetin intakes by men and women were 16.2 and 13.7 mg day^−1^, and 19.6 and 17.3 mg day^−1^, respectively. The daily quercetin intakes of men and women were not significantly different as well (Figure 4b). Quercetin intake during winter did not show a correlation with age for both men and women (Figure 4c). During winter, quercetin was mainly ingested from onions (Figure 3b).

## 4. Discussion

In this study, onions and green tea were shown to be major sources of quercetin intake both in summer and in winter. The quercetin contents of the edible part of onions in Japan were 10–50 mg 100 g^−1^ fresh weight. Onions grown in Hokkaido had been reported to contain 30–50 mg quercetin (100 g)^−1^ fresh weight [25]. The principal cultivar “Kita momiji 2000” contained approximately 40 mg quercetin (100 g)^−1^ [24]. From autumn to spring, the onions grown in the major production area of Hokkaido were eaten throughout Japan. On the other hand, the quercetin content of the green tea infusion was less than that of onions. In our study, the green tea infusion contained 2.1 mg quercetin 100 mL^−1^, whereas it had been previously reported to contain 0.11 mg or 4.23 mg quercetin 100 g^−1^ [26,27]. Because onion and green tea are the most common vegetable and beverage, they appear to be the major food sources of quercetin in Japan.

Epidemiological studies showed that moderate wine consumption reduced the risk of cardiovascular diseases [28]. Consumption of wine rich in polyphenols is expected to have health benefits [29]. Yoo *et al.* reported the positive correlation for quercetin concentration (0.1–1 mg L^−1^) with total phenols and antioxidant activity in red wines [30]. However, the result of our survey on alcohol drinking showed that about 3% and 6% of men and women were habitual wine drinkers, respectively. Wine consumption probably does not contribute to daily quercetin intake in this cohort.

A significant amount of quercetin was consumed from vegetables, such as asparagus, green pepper, tomato, and red leaf lettuce during summer. The results of the two-day weighted food record by eight volunteers also showed that asparagus, green peppers, tomatoes, and red leaf lettuce are also major food sources of quercetin. We showed that cooked asparagus and green pepper maintained a substantial concentration of quercetin. These vegetables, which are in season during summer, contributed more to quercetin intake in summer than that in winter.

Although the contribution percentages for different foods varied, the estimated daily quercetin intake by residents in summer we comparable to that in winter. The results of the FFQ by 570 subjects showed that the estimated quercetin intakes by men were lower than those by women. The daily quercetin intakes by the subjects showed a low correlation with age in both men and women. On the other hand, the quercetin intakes by men and women were not significantly different and the daily quercetin intakes were not correlated with age in winter. This difference may have occurred because there were fewer subjects in the survey during winter.

There have been several reports on the estimated daily quercetin intake by Japanese women but not by men [26,27,31]. The simple FFQ, which asked about the frequency and portion size of 15 commonly-eaten and quercetin-rich food items, enabled estimation of the quercetin intake by men and women, including older people. Although the quercetin content of plant foods differs depending on the cultivars or cultivation conditions, quercetin intakes were estimated using a database established in other country or other period in other epidemiological studies on quercetin and risks of lifestyle-related diseases [1,2,3,4,5,6,7]. In this study we precisely estimated the quercetin intake by subjects in a cohort study and found that partial correlation analysis adjusted for age showed that quercetin intake was negatively correlated with diastolic blood pressure (rpar = −0.145, *p* = 0.008). Edwards *et al.* showed that supplementation with 730 mg quercetin day^−1^ for 28 days reduced systolic and diastolic blood pressure in stage 1 hypertensive patients [32]. Egert *et al.* showed that supplementation of quercetin reduced systolic blood pressure in overweight–obese carriers of the apo ε3/ε3 genotype [33]. Quercetin intake may contribute in decreasing the levels of blood pressure. Arai *et al*. showed that after adjustment for age, body mass index, and total energy intake, the quercetin intake was inversely correlated with the plasma total cholesterol and LDL cholesterol concentrations in Japanese women [26]. Although the cross-sectional analysis did not show any other significant correlation, further prospective study will be able to elucidate the causal association between quercetin intake and health indexes or risks of lifestyle related diseases.

The antioxidant activity is thought to be a prevention mechanism of lifestyle-related diseases. Although Edward *et al.* did not find the effect of supplemented quercetin on oxidative stress indices in hypertensive subjects [32], Egert *et al.* reported that the supplementation of quercetin at a dose of 150 mg day^−1^ for 6 weeks reduced the oxidized LDL concentration in overweight subjects [34]. Terao *et al.* recently showed that plasma quercetin metabolites detected after combined intake of sautéed onion and tofu were different from those detected after the intake of sautéed onion in healthy volunteers [35]. The antioxidant effect of quercetin depends on the matrix that is it found in the intake of other foods and other factors. Evaluation of the antioxidant effect of the quercetin-rich foods and the foods in diet may help to elucidate the preventive effect of quercetin on lifestyle-related diseases.

The averages of the estimated daily quercetin intake by residents of Sobetsu in Hokkaido were 16.2 mg day^−1^ in summer and 18.3 mg day^−1^ in winter. Arai *et al*. reported that the average estimated quercetin intake was 9.3 mg day^−1^ using a three-day weighted dietary record by women living in the northern part of Japan [26]. Ioku *et al*. estimated the average quercetin intake by middle-aged and elderly women living in the Kansai area in Japan as 17.8 mg day^−1^ [27]. Otaki *et al*. estimated the average intake of quercetin by women in the northern part of Japan as 15.8 mg day^−1^ with a cross-sectional study using a 24 h weighted dietary record [31]. Our results are similar to the results of these previous studies conducted 10–20 years ago.

## 5. Conclusions

In conclusion, we have estimated daily quercetin intake by residents, who are the subjects of the longitudinal cohort study, in a town in Hokkaido. The average and median daily quercetin intake using FFQ was 16.2 and 15.5 mg day^−1^, respectively, during summer. The results were similar to the estimated quercetin intake by Japanese women in the previous studies. The quercetin intake by men was lower than that by women. The quercetin intakes showed a low correlation with age in both men and women. The daily intake of quercetin, which was mainly provided by onions and green tea, was comparable in summer and in winter. Summer vegetables, such as asparagus, green pepper, tomatoes, and red leaf lettuce, were also good sources of quercetin, which is a promising food component for the prevention of lifestyle-related diseases. Further prospective study will probably be able to elucidate the causal association between quercetin intake and health indexes or risks of lifestyle-related diseases.

## References

[1] Formica J.V., Regelson W. (1995). Review of the biology of quercetin and related bioflavonoids. Food Chem. Toxicol..

[2] Knekt P., Jarvinen R., Reunanen A., Maatela J. (1996). Flavonoid intake and coronary mortality in Finland: A cohort study. BMJ.

[3] Arts I.C.W., Hollman P.C.H. (2005). Polyphenols and disease risk in epidemiologic studies. Am. J. Clin. Nutr..

[4] Hollman P.C.H., Katan M.B. (1999). Dietary flavonoids: Intake, health effects and bioavailability. Food Chem. Toxicol..

[5] Hooper L., Kroon P.A., Rimm E.B., Cohn J.S., Harvey I., Le Cornu K.A., Ryder J.J., Hall W.L., Cassidy A. (2008). Flavonoids, flavonoid-rich foods, and cardiovascular risk: A meta-analysis of randomized controlled trials. Am. J. Clin. Nutr..

[6] Kelly G.S. (2011). Quercetin. Monograph. Altern. Med. Rev..

[7] Peterson J.J., Dwyer J.T., Jacques P.F., McCullough M.L. (2012). Associations between flavonoids and cardiovascular disease incidence or mortality in European and US populations. Nutr. Rev..

[8] Terao J., Kawai Y., Murota K. (2008). Vegetable flavonoids and cardiovascular disease. Asia Pac. J. Clin. Nutr..

[9] Morales J., Gunther G., Zanocco A.L., Lemp E. (2012). Singlet oxygen reactions with flavonoids. A theoretical-experimental study. PLoS ONE.

[10] Lagoa R., Graziani I., Lopez-Sanchez C., Garcia-Martinez V., Gutierrez-Merino C. (2011). Complex I and cytochrome c are molecular targets of flavonoids that inhibit hydrogen peroxide production by mitochondria. Biochim. Biophys. Acta.

[11] Mahesh T., Menon V.P. (2004). Quercetin allievates oxidative stress in streptozotocin-induced diabetic rats. Phytother. Res..

[12] Boesch-Saadatmandi C., Wagner A.E., Wolffram S., Rimbach G. (2012). Effect of quercetin on inflammatory gene expression in mice liver *in vivo*—Role of redox factor 1, miRNA-122 and miRNA-125b. Pharmacol. Res..

[13] Kobori M., Masumoto S., Akimoto Y., Takahashi Y. (2009). Dietary quercetin alleviates diabetic symptoms and reduces streptozotocin-induced disturbance of hepatic gene expression in mice. Mol. Nutr. Food Res..

[14] Kobori M., Masumoto S., Akimoto Y., Oike H. (2011). Chronic dietary intake of quercetin alleviates hepatic fat accumulation associated with consumption of a Western-style diet in C57/BL6J mice. Mol. Nutr. Food Res..

[15] Dong J., Zhang X., Zhang L., Bian H., Xu N., Bao B., Liu J. (2014). Quercetin reduces obesity-associated ATM inflammation in mice: A mechanism including AMPKα1/SIRT1. J. Lipid Res..

[16] Murota K., Hotta A., Ido H., Kawai Y., Moon J.H., Sekido K., Hayashi H., Inakuma T., Terao J. (2007). Antioxidant capacity of albumin-bound quercetin metabolites after onion consumption in humans. J. Med. Investig..

[17] Ishisaka A., Kawabata K., Miki S., Shiba Y., Minekawa S., Nishikawa T., Mukai R., Terao J., Kawai Y. (2013). Mitochondrial dysfunction leads to deconjugation of quercetin glucuronides in inflammatory macrophages. PLoS ONE.

[18] Zamora-Ros R., Knaze V., Lujan-Barroso L., Slimani N., Romieu I., Fedirko V., de Magistris M.S., Ericson U., Amiano P., Trichopoulou A. (2011). Estimated dietary intakes of flavonols, flavanones and flavones in the European Prospective Investigation into Cancer and Nutrition (EPIC) 24 hour dietary recall cohort. Br. J. Nutr..

[19] Ioku K., Aoyama Y., Tokuno A., Terao J., Nakatani N., Takei Y. (2001). Various cooking methods and the flavonoid content in onion. J. Nutr. Sci. Vitaminol..

[20] Ohnishi H., Saitohi S., Takagii S., Katohi N., Chibai Y., Akasakai H., Nakamura Y., Shimamoto K. (2006). Incidence of type 2 diabetes in individuals with central obesity in a rural Japanese population: The Tanno and Sobetssu study. Diabetes Care.

[21] Mitsumata K., Saitoh S., Ohnishi H., Akasaka H., Miura T. (2012). Effects of parental hypertension on longitudinal trends in blood pressure and plasma metabolic profile: Mixed-effects model analysis. Hypertension.

[22] Slimestad R., Fossen T., Vagen I.M. (2007). Onions: A source of unique dietary flavonoids. J. Agric. Food Chem..

[23] Jin J., Koroleva O.A., Gibson T., Swanston J., Magan J., Zhang Y., Rowland I.R., Wagstaff C. (2009). Analysis of phytochemical composition and chemoprotective capacity of rocket (*Eruca sativa* and *Diplotaxis tenuifolia*) leafy salad following cultivation in different environments. J. Agric. Food Chem..

[24] Watanabe J., Takebayashi J., Takano-Ishikawa Y., Yasui A. (2012). Evaluation of a Method to Quantify Quercetin Aglycone in Onion (*Allium cepa*) by Single- and Multi-laboratory Validation Studies. Anal. Sci..

[25] Okamoto D., Noguchi Y., Muro T., Morishita M. (2006). Genetic variation of quercetin glucoside content in onion (*Allium cepa* L.). J. Jpn. Soc. Hortic. Sci..

[26] Arai Y., Watanabe S., Kimira M., Shimoi K., Mochizuki R., Kinae N. (2000). Dietary intakes of flavonols, flavones and isoflavones by Japanese women and the inverse correlation between quercetin intake and plasma LDL cholesterol concentration. J. Nutr..

[27] Ioku K., Okuda T., Higuchi H., M. K., Takei Y. (2008). Investigation of the Flavonoid Intake in a Daily Meal of the Kansai in the Middle-aged women. Osaka Kyoiku Univ. Repos. II.

[28] Arranz S., Chiva-Blanch G., Valderas-Martínez P., Medina-Remón A., Lamuela-Raventós R.M., Estruch R. (2012). Wine, beer, alcohol and polyphenols on cardiovascular disease and cancer. Nutrients.

[29] Yoo Y.J., Saliba A.J., MacDonald J.B., Prenzler P.D., Ryan D. (2013). A cross-cultural study of wine consumers with respect to health benefits of wine. Food Qual. Prefer..

[30] Yoo Y.J., Prenzler P.D., Saliba A.J., Ryan D. (2011). Assessment of some Australian red wines for price, phenolic content, antioxidant activity, and vintage in relation to functional food prospects. J. Food Sci..

[31] Otaki N., Kimira M., Katsumata S., Uehara M., Watanabe S., Suzuki K. (2009). Distribution and major sources of flavonoid intakes in the middle-aged Japanese women. J. Clin. Biochem. Nutr..

[32] Edwards R.L., Lyon T., Litwin S.E., Rabovsky A., Symons J.D., Jalili T. (2007). Quercetin reduces blood pressure in hypertensive subjects. J. Nutr..

[33] Egert S., Boesch-Saadatmandi C., Wolffram S., Rimbach G., Muller M.J. (2010). Serum lipid and blood pressure responses to quercetin vary in overweight patients by apolipoprotein E genotype. J. Nutr..

[34] Egert S., Bosy-Westphal A., Seiberl J., Kurbitz C., Settler U., Plachta-Danielzik S., Wagner A.E., Frank J., Schrezenmeir J., Rimbach G. (2009). Quercetin reduces systolic blood pressure and plasma oxidised low-density lipoprotein concentrations in overweight subjects with a high-cardiovascular disease risk phenotype: A double-blinded, placebo-controlled cross-over study. Br. J. Nutr..

[35] Nakamura T., Murota K., Kumamoto S., Misumi K., Bando N., Ikushiro S., Takahashi N., Sekido K., Kato Y., Terao J. (2014). Plasma metabolites of dietary flavonoids after combination meal consumption with onion and tofu in humans. Mol. Nutr. Food Res..

